# ELECTROMAGNETIC FIELDS: Conference, Hearing Call Up Cell Phone Use

**DOI:** 10.1289/ehp.117-a486

**Published:** 2009-11

**Authors:** Kellyn S. Betts

**Affiliations:** **Kellyn S. Betts** has written about environmental contaminants, hazards, and technology for solving environmental problems for publications including *EHP* and *Environmental Science & Technology* for more than a dozen years

Although experts agree more research is needed to investigate whether cell phone use can cause brain cancers, especially in children, a group of scientists, physicians, and public health specialists around the world are urging that simple precautionary measures be taken just in case. Environmental groups have also stepped up pressure for regulatory action, but very little has taken place in the United States to date. The widespread use of cell phones throughout the world makes questions about their safety “one of the most important matters that we have to deal with in public health today,” says Devra Davis, a professor of epidemiology at the University of Pittsburgh’s Graduate School of Public Health and co-organizer of a conference on the topic held in Washington, DC, in September 2009.

There were an estimated 277 million cell phone users in the United States as of June 2009, according to the industry group CTIA–The Wireless Association®, and about 4.1 billion worldwide, according to the United Nations. “With so many users, [cell phones] could translate into a significant public health problem should their use even slightly increase the risk of adverse health effects,” said John Bucher, associate director of the National Toxicology Program, at a hearing held during the conference by the Senate Labor, Health and Human Services, Education and Related Agencies Appropriations subcommittee.

Bucher also said the configuration of children’s skulls allows cell phone radiation—which falls between FM radio and microwaves on the electromagnetic spectrum—to penetrate deeper into the brain. In areas such as Africa and Brazil the majority of users are very young, Davis says. However, although some researchers believe children receive a higher dose of radiation from cell phone use, no studies published to date have evaluated their risk.

In one of the most recent reports on the issue, a review of case–control studies comparing brain tumors in cell phone users versus non-users showed a slightly elevated risk of acoustic neuroma and glioma in people who began using cell phones as adolescents. Risk was highest for ipsilateral tumors—those occurring on the same side of the head that patients reported typically holding their phones—although such retrospective measures are notoriously difficult to confirm. The review was published in the August 2009 issue of *Pathophysiology* by Lennart Hardell and colleagues.

Hardell’s findings are supported by a meta-analysis published 13 October 2009 ahead of print in the *Journal of Clinical Oncology*, in which Seung-Kwon Myung and colleagues evaluated 23 epidemiologic studies and found that the 8 most rigorous studies showed an elevated risk of tumors in cell phone users compared with people who rarely or never used the phones. The risk was highest among those who had used cell phones for 10 years or more.

But assessments by the World Health Organization, the U.S. Food and Drug Administration (FDA), and technical organizations such as the Institute of Electrical and Electronics Engineers have concluded the available evidence does not demonstrate that wireless phones cause health effects, says Linda Erdreich, senior managing scientist at the consultancy Exponent, who testified at the Senate hearing at the request of CTIA. The FDA, one of two agencies that share regulatory authority over U.S. cell phones, states on its website that “The scientific evidence does not show a danger to any users of cell phones from [radiofrequency] exposure, including children and teenagers,” but nevertheless describes ways for cell phone users to reduce their potential exposure to radiation.

Dariusz Leszczynski, head of the radiobiology laboratory at Finland’s Radiation and Nuclear Safety Authority, stresses that the currently available scientific research about the effects of radiation emitted by cell phones is contradictory. He says, “In each area of investigation, there are studies showing effects and studies showing no effect [but ] it is premature . . . to say that the use of mobile phones is safe.” Accordingly, the health agencies of Finland, France, Germany, Israel, Switzerland, and the United Kingdom have issued precautionary advisories regarding cell phone use for consumers of all ages.

When considered together, the research to date gives “a hint that something is going on,” particularly for people who have used cell phones for more than 10 years, but more research is needed on more people to tell for sure, according to Siegal Sadetzki, head of cancer and radiation epidemiology at the Sackler School of Medicine’s Gertner Institute in Tel Aviv, Israel. She headed up a study published in the 15 February 2008 issue of the *American Journal of Epidemiology* that reported an elevated risk of ipsilateral parotid gland tumors among cell phone users who spent the most time on the phone.

Sadetzki’s work was part of the International Agency for Research on Cancer’s INTER-PHONE study, which evaluated whether cell phone use can increase cancer risk. The study concluded in 2006, but although many of the individual components have reported results, a combined analysis still has not been published.

Given that the widespread use of cell phones did not begin until 1995, and based on observations of brain tumor growth in survivors of the Japanese atom bombings, any brain cancers linked to cell phone use wouldn’t be expected to become evident until at least 2032, Sadetzki says. The fact that any studies have found an association between cell phone use and such tumors is, she says, worrisome.

New research projects under way to help shed light on cell phones’ health risks include the Cohort Study of Mobile Phone Use and Health, which began earlier this year. This prospective cohort study will be conducted in Europe to assess cell phone use in relation to a variety of health effects, including brain tumors, blood cancers, and neurodegen erative and cerebrovascular diseases. The MOBI-KIDS case–control study, which will follow 1,900 people aged 10–24 years with brain tumors and a similar number without brain tumors for five years, is expected to begin recruiting next year.

To increase the amount of toxicologic data on the subject, the National Toxicology Program has begun a series of studies using special reverberation chambers to evaluate the effects on rodents of exposure to cell phone radiation. Rodents will be exposed for up to 20 hr a day to the same modulations and frequencies of cell phone radiation currently experienced in the United States. Results are expected in 2014.

“Until scientists have much more information about cell phone radiation, it’s smart for consumers to buy phones with the lowest emissions,” says Olga Naidenko, a senior scientist for the Environmental Working Group. She also recommends that people use a headset or speakerphone and substitute text messages for conversations when possible, and that children’s cell phone use be limited.

## Figures and Tables

**Figure:**
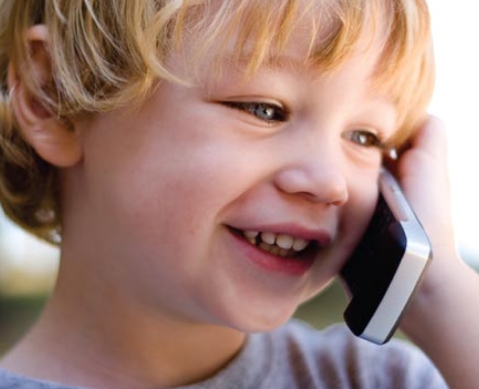
While public health experts debate the science of cell phone safety, parents debate how young is too young to have one of the devices.

